# Fear-avoidance beliefs are associated with exercise adherence: secondary analysis of a randomised controlled trial (RCT) among female healthcare workers with recurrent low back pain

**DOI:** 10.1186/s13102-020-00177-w

**Published:** 2020-05-04

**Authors:** Annika Taulaniemi, Markku Kankaanpää, Marjo Rinne, Kari Tokola, Jari Parkkari, Jaana H. Suni

**Affiliations:** 1 UKK Institute for Health Promotion Research, Tampere, Finland; 2 Department of Physical and Rehabilitation Medicine, Tampere University Hospital, Tampere, Finland

**Keywords:** Exercise compliance, Neuromuscular exercise, Pilates, Fear of pain, Lumbar pain, Exercise intervention study, Nursing

## Abstract

**Background:**

Exercise is recommended for the treatment and management of low back pain (LBP) and the prevention of chronicity. Exercise adherence has been only modest in intervention studies among people with musculoskeletal pain. Fear-avoidance beliefs (FABs) are known to affect exercise adherence.

**The purpose** was twofold: to examine which bio-psycho-social factors contributed to exercise adherence during a 6-month neuromuscular exercise intervention among female healthcare workers with recurrent LBP, and to investigate how exercising affects FABs at 6 and 12 months’ follow-up.

**Methods:**

Some 219 healthcare workers aged 30–55 years with mild-to-moderate re-current non-specific LBP were originally allocated into: 1) exercise, 2) counselling, 3) combined exercise and counselling, and 4) control groups. In the present secondary analysis, groups 1 and 3 (exercise only and exercise+counselling) were merged to be exercisers and groups 2 and 4 were merged to be non-exercisers. Baseline variables of the exercise compliers (≥24 times over 24 weeks; *n* = 58) were compared to those of the non-compliers (< 1 time/week, 0–23 times; *n* = 52). The effects of the exercise programme on FABs were analysed by a generalised linear mixed model according to the intention-to-treat principle (exercisers; *n* = 110 vs non-exercisers; *n* = 109) at three measurement points (baseline, 6, and 12 months). A per-protocol analysis compared the more exercised to the less exercised and non-exercisers.

**Results:**

A low education level (*p* = 0.026), shift work (*p* = 0.023), low aerobic *(p* = 0.048) and musculoskeletal (*p* = 0.043) fitness, and high baseline physical activity-related FABs (*p* = 0.019) were related to low exercise adherence. The exercise programme reduced levels of both physical activity- and work-related FABs, and there was a dose response: FABs reduced more in persons who exercised ≥24 times compared to those who exercised 0–23 times.

**Conclusion:**

Healthcare workers who had lower education and fitness levels, worked shifts, and had high physical activity-related FABs had a lower adherence to the 6-month neuromuscular exercise programme. Exercising with good adherence reduced levels of FABs, which have been shown to be linked with prolonged LBP. Motivational strategies should be targeted at persons with low education and fitness levels and high FABs in order to achieve better exercise adherence.

## Background

Low back pain (LBP) is common among people in all ages, but disability from LBP is highest in working-age groups [1]. Among healthcare workers, LBP is the leading musculoskeletal disorder [2] and it has been reported to be the most costly and common self-reported disease [3]. Major contributors to the high incidence of LBP among healthcare workers are physically heavy nursing duties, such as lifting and transferring patients, and working in awkward positions [2, 4, 5].

In physically demanding work duties, maintaining a healthy back requires adequate aerobic and musculoskeletal fitness level [6–8]. Low ratings of self-reported physical capacity have been shown to be a predictor for future LBP in female healthcare workers [9]. Female nurses with a recent back injury also show more impairment in lumbar movement control [10], which has also been suggested to play an important role in maintaining a healthy back [11, 12]. A systematic review on efficacy of interventions for LBP in nursing personnel [13] revealed no strong evidence of efficacy for any intervention in preventing or treating LBP in healthcare workers.

Exercise is the most often recommended treatment for increasing fitness levels [14], lumbar movement control [12], and for the management and prevention of LBP [15–19]. Thus, in the management of spinal pain exercise adherence is important to realise the beneficial effects of exercising. Adherence is a key link between the process and outcome of exercise interventions among people with musculoskeletal pain, and poor exercise adherence compromises the effectiveness of treatment [20].

Barriers to exercise adherence, such as pain with exercise [21], fear of movement and pain aggravation [22], low self-efficacy [21], psychological dysfunction [21], poor social support [21], lack of time [23], and uncertainty about the benefits of exercise [24], have been reported among people with musculoskeletal pain [21, 22, 24, 25]. Aforementioned studies have been conducted among older adults [25], people with chronic LBP [26] or other chronic musculoskeletal pain [22, 24] or among people receiving physiotherapy [21]. To our knowledge, similar studies which concern exercise adherence among people with heavy physical work and non-chronic LBP have not been conducted.

Fear avoidance is the belief that activities should be avoided to reduce pain [27]. Fear of pain develops as a result of a cognitive interpretation of nociseption as something threatening, and this fear affects attention processes (hypervigilance) and leads to the avoidance of behaviours, like physical activity and exercise, which are expected to cause pain [27]. Fear-avoidance beliefs (FABs) influence treatment effects and are prognostic to poor outcomes in subacute LBP [28]. Cognitive behavioural therapy is recommended to reduce FABs among people with LBP [29, 30], but effects of exercise interventions on FABs among people with sub-acute or recurrent LBP are less clear.

Besides FABs, there are probably several other internal factors (like a lack of interest [31], and low self-efficacy [32]) that compromise adherence to exercise among people with LBP. Exercise might be avoided, because it is believed to cause pain, one is uncertain about the benefits of exercising, and one’s capabilities to perform and manage the instructed exercises [32]. If those internal factors are combined with external barriers (like lack of time, environment, and transfer), exercise adherence might be challenging.

Among people with chronic LBP, improvements in physical functioning are more strongly associated with adherence to exercise than with the type of exercise [26]. Some 50–70% of people with chronic LBP are non-adherent to prescribed home exercise [33]. Thus, factors associated with adherence to exercise in LBP require more investigation [33]. Exercise adherence among people with musculoskeletal pain in general is a poorly studied subject [20].

In a previously reported study, we found that a 6-month modified Pilates-type neuromuscular exercise intervention, which focused on controlling the neutral lumbar spine posture and developing the muscle strength and endurance needed in heavy nursing tasks, was effective in reducing lumbar pain and lumbar movement control impairments among a sample of nursing personnel with sub-acute or recurrent LBP [34]. In the 6-month intervention, the target was to exercise twice a week, i.e. 48 times in 24 weeks. Exercise adherence was only modest; the mean attendance rate was 26.1 (of the targeted 48). The reduction of LBP intensity and lumbar movement control impairment was significantly better in those who exercised once a week or more compared to the less exercised and non-exercisers [34]. Thus, factors affecting exercise adherence needed more investigation. Our hypothesis was that pain intensity, psychological, and environmental factors compromise exercise adherence, and that the modified Pilates-type exercise reduces FABs-PA.

The purpose of this study was to (1) investigate the effects of baseline bio-psychosocial factors on exercise adherence among female healthcare personnel with sub-acute or recurrent LBP. Furthermore, we sought to (2) examine the effects of the exercise intervention on the development of FABs over time (at 6 and 12 months’ follow-up).

## Methods

### Study design

This study is based on the data of a four-arm randomised controlled trial (RCT) among female healthcare personnel (NURSE-RCT, clinical trial registration NCT01465698) [35], in which healthcare personnel with sub-acute or recurrent LBP were randomised to participate in neuromuscular exercise/non-exercise and to receive/not receive back care counselling for 6 months [36]. In the secondary analysis, those receiving exercise (combined exercise + counselling, and exercise only) were merged to be exercisers, and non-exercisers (counselling only and controls) were merged to be the controls [34].

The study was conducted in the form of three identical consecutive sub-studies. The participants were female healthcare workers in physically demanding duties: in old people’s homes and geriatric wards (in the first sub-study in 2011, *n* = 56); in home service, public healthcare units, and community hospital wards (in the second sub-study in 2012; *n* = 80); and in university hospital wards (in the third sub-study in 2013; *n* = 83) in the city of Tampere, Finland. The protocol and time frame of each sub-study are presented in the study protocol [35]. The recruitment of participants, eligibility criteria, and reasons for exclusion have been previously described in detail [36]. Briefly, 30–55-year-old female healthcare workers were eligible if they had worked in their current job for at least 12 months and had experienced LBP of an intensity of 2 or above on a numeric rating scale (NRS; 0–10) [37] during the previous 4 weeks. The exclusion criteria were a specific or serious earlier back condition (disc protrusion, fracture, surgery), chronic LBP (pain duration ≥7 months), pregnancy or recent delivery (< 12 months), and engagement in a neuromuscular-type exercise (NME) more than once a week.

The power calculations, recruitment process, randomisation, and ethical issues of the NURSE-RCT have been presented previously [35], as have the contents of the exercise intervention [34].

**Fig. 1:**
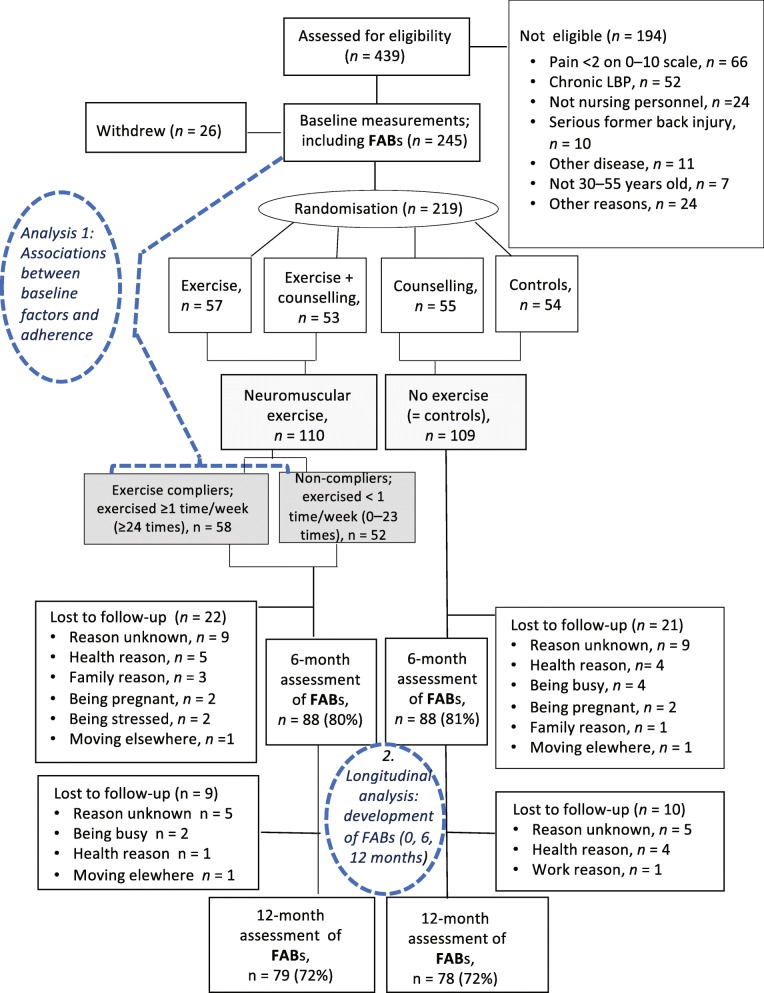
Trial profile (CONSORT flow chart). Footnot to Fig. 1: Analysis methods for studying (1) the associations between baseline factors and exercise adherence rate, and (2) the effects of the exercise intervention on fear-avoidance beliefs (FABs)

The study design and flow of the participants are shown in Fig. 1.

### Participants

The participants were female healthcare workers who engaged in physically demanding work (including lifting, patient transfer and working in awkward positions) and suffered from sub-acute or recurrent LBP. The mean age of the participants was 46 years, and they had worked in their current job on average for 11 years [7]. Some 87% were nurses or nursing assistants, and 70% did shift work [7]. In the pre-study screening, most of the study subjects (82%) experienced LBP on a few or most days of the week, but not daily, while 18% had LBP daily [7]. At the baseline, the mean of the pain intensity measured on a visual analogue scale (VAS; 0–100) [38] during the previous 4 weeks was 36.2 (SD 22.6) [7]. The majority (77%) of the study sample can be described as having sub-acute, mild-to-moderate, recurrent or fluctuating non-specific LBP (4). Among those with daily pain (18%), the pain intensity was higher the mean in VAS being 55.7 (25.3).

### Measurements

A wide range of measurements was taken at the baseline. In addition to background factors (age, education level, marital status, occupation, number of working years in the current job, working hours, smoking, perceived health and perceived fitness in comparison to persons of the same age and gender, current use of medication, high blood pressure (yes/no), and hormonal status); LBP intensity (VAS; 0–100) during the previous 4 weeks [38]; the frequency of LBP; the number of musculoskeletal pain sites [7]; quality of life (RAND 36) with eight sub-scales [39, 40]; depression (using the modified Finnish version of the Patient Health Questionnaire; PHQ-9) [41]; the short form of the workability index [42]; physical functioning in nursing tasks [35]; tiredness, sleepiness, and difficulties in recovering from work [43]; work-induced exertion in different body parts [44]; and psychosocial factors at work (Finnish work satisfaction questionnaire) [45] were investigated by questionnaires. FABs were measured with a questionnaire assessing FABs related to work (FAB-W) and physical activity (FAB-PA) [46]. Three questions considering long-term sick leave were removed from the original FABs questionnaire, because the participants were still in work [35].

Physical measurements included body mass index (BMI), movement control of the low back [47, 48], and performance tests for physical fitness, namely aerobic fitness by the 6-min walk test [49], muscular strength (modified push-up [50], one-legged squat with progressively increasing external load (10% of body weight after each performance up to 40%) [50], vertical jump [50], modified sit-ups [51]), agility by running a figure-of-eight [52], flexibility by trunk lateral side bending [50], and rhythm coordination [52]. More precise information on the measurements is given in the study protocol article [35], the article on the repeatability of the physical measurements [48], and the baseline analysis of the study sample [7].

### Exercise interventions in the NURSE-RCT

The contents of the 6-month exercise intervention have been described previously [34]. The modified 6-month Pilates-type exercise intervention programme, which focused on controlling the neutral spine posture, started with light and easier exercises, and it was progressive in terms of demands for coordination, balance, and muscular strength over three stages. The goal was to exercise twice a week; during the first 2 months (stage I) in supervised neuromuscular exercise (NME) classes (lasting 60 min) and during the next 4 months (stages II and III) in one supervised class and one home session with the help of a DVD (lasting 50 min) or booklet produced for the study [34]. During stages II and III, the participants were also allowed to exercise in supervised group sessions more than once a week if exercise at home was inconvenient, and also only at home if the group sessions were difficult to attend. During the progression (stages II and III), the participants were allowed and/or advised to do easier exercises from the previous stage if the more challenging exercises proved too demanding.

The leaders of the neuromuscular exercise groups were all certified Pilates instructors with a background in physiotherapy, a master’s degree in health sciences, or both [34]. Supervised exercise groups were organised in facilities near the workplaces of the healthcare personnel. Group sessions were provided on weekdays starting 15 min after the typical work shifts ended. The exercise classes, videos, and booklets were free for the participants, but they exercised in their own time [36].

### Adherence to exercise

The instructors monitored the participation of supervised group exercise, and study subjects kept an exercise diary of their home practice. The structured exercise diaries were returned at the end of stage II (week 16) and stage III (week 24). Attendance of the supervised exercise sessions and the number of home exercise sessions were added together to determine the total exercise attendance rate.

### Motivational strategies

All participants in the exercise group received an information letter at the beginning of the exercise intervention about the goals and principles of the exercise programme. During the 4th week of the first-stage exercise period, those who had not participated in any group-based exercise sessions received a telephone call from a research nurse (not involved in the exercise intervention or measurements), who encouraged them to start to exercise. All participants received two material packages (between stages I and II, and between stages II and III), which included an exercise DVD, exercise booklet, exercise diary for home practice, and a letter including information about the study and the importance of regular exercise. They also received two e-mails during stage II in order to encourage exercise, and a letter before the 6-month follow-up measurements from the principal investigator (JS).

To avoid any contamination to the back care counselling intervention (in the original four-arm setting), and to ensure exactly the same information to all who were allocated to the exercise group, the exercise instructors focused on instructing the standardised exercise programme (individual modifications due to musculoskeletal problems other than LBP were allowed). All other kinds of counselling (e.g. lifestyle, pain management, and ergonomics) were avoided in the exercise classes.

### Statistical methods

Power calculations (at least 160 subjects needed) for the original NURSE-RCT have been reported previously [35], as has the randomisation of the participants [36].

Partial correlation analysis was conducted between all background and baseline variables and the adherence rate to determine which of the 60 different variables could have an association with the exercise adherence. Those variables showing a statistically significant association with the exercise adherence rate were selected for bivariate analysis with the adherence rate; the analytical methods were Spearman’s correlation for continuous variables and the Kruskall–Wallis test for the categorical variables.

The median (24 times) was used to split the exercise group into the compliers (those who exercised once a week or more; ≥24 times during the 24 weeks) and non-compliers (those who exercised 0–23 times) We examined the baseline characteristics of the participants randomised to the exercisers by the adherence status for those variables showing statistically significant associations with the adherence rate in the bivariate analysis. The analytical methods were the independent samples *t*-test, the χ^2^ test, or the Mann–Whitney *U* test as applicable.

To analyse the effects of the exercise programme on FABs, the mean differences in time (at three measurement points: baseline, 6 months, and 12 months) between the two groups (exercisers vs non-exercisers) were tested using a generalised linear mixed model (GLMM) (Fig. 1). To take the interaction between back counselling and exercise into consideration, all analyses were first adjusted for counselling. Second, the sub-study was included as a random effect in all the GLLM analysis models to indicate the possible heterogeneity between the study sites and study time in the three consecutive sub-studies. Other confounding factors were background variables (age, civil status, education), work-related factors (shift work/regular work, psycho-social factors at work [45], perceived work-induced lumbar exertion [44]), and health-related factors (BMI, hormonal status, perceived health, perceived fitness, blood pressure, current medication, self-reported physical activity and fitness components). Only those confounding factors that improved the model in the second stage in the sense of Bayesian information criteria were included in the final adjusted model.

After analysis according to the intention-to-treat (ITT) principle (Fig. 1), the study sample was assigned into two groups in order to investigate the effectiveness of the exercise on FABs, based on a per-protocol (PP) analysis. The mean difference in time (0, 6, 12 months) of exercise compliers (≥24 exercise sessions) were estimated and compared to the results of a combined group of non-compliers and non-exercisers (0–23 exercise sessions + controls).

The correlation between the change in LBP intensity from the baseline to 6 months [34] and the change in the results of the FAB measurements after the intervention period were calculated by Spearman’s correlation coefficient (r_s_). Associations between professional status and fear avoidance at the baseline were analysed by analysis of variance (ANOVA).

All the analyses were conducted using SPSS (IBM Corp. Released 2017. IBM SPSS Statistics for Windows, Version 25.0 Armonk, NY: IBM Corp.).

## Results

### Exercise adherence

The target was for the participants to exercise twice a week – i.e. 48 sessions over 24 weeks. The mean attendance rate was 26.3 (12.2) exercise sessions. Some 53% of the participants exercised 1–2 times/week. The mean attendance rate was 1.1 times/week during the whole intervention. During the last 8 weeks, the mean attendance rate of the group-based exercise decreased, but the home-based exercise rate increased (total amount remaining 1.1 times /week). Only two people out of the 110 exercised regularly twice a week during the 6-month intervention period.

Of those who were allocated to the exercise group, 10% did not exercise at all, and another 10% took part in only 1–5 exercise sessions in the 6-month period. Of the whole study sample (*n* = 219), 80% (*n* = 176) and 72% (*n* = 157) participated in the 6-month and 12-month follow-up measurements, respectively [36]. At 6 months, 22 persons had dropped out, and 91% of them (*n* = 20) belonged to the least exercised group (0–5 exercise sessions). At 6 months, the dropout rate (*n* = 21) was equal among non-exercisers (Fig.1).

### Baseline variables associated with exercise adherence

The bivariate associations between exercise attendance rates and continuous background and baseline variables is presented in Table 1. The associations for categorical variables are shown in Table 2. Between-group differences for those variables that had statistically significant correlations with adherence rates in the bivariate analysis are presented in Table 3 for the exercise compliers vs non-compliers.

**Table 1:** Bivariate correlation between baseline continuing variables and exercise adherence rate

	Correlation with adherence (r_s_)	Missing	*p*-value
Running figure-of-eight	−0.27	9	0.006
One-legged squat	0.19	2	0.048
6MWT	0.28		0.003
Quality of life			
Physical functioning	0.19	4	0.045
Energy	0.15	4	0.12
Social functioning	0.18	4	0.06
General health	0.23	4	0.019
Workability index	0.26		0.006
Depression; PHQ-9	−0.20	1	0.038
Musculoskeletal exertion	0.25	2	0.009
FABs (total)	−0.26	7	0.009
FAB-PA	−0.32	1	0.001
Intensity of LBP	−0.06	2	0.54

6MWT = 6-min walk test, *FAB* fear-avoidance beliefs, *FAB-PA* fear-avoidance beliefs related to physical activity, *LBP* low back pain, *PHQ-9* Patient Health Questionnaire, 9 items

**Table 2:** Association between baseline categorical variables and exercise adherence rate, analysed by the Kruskall–Wallis test

	*n*	Exercise adherence; median	Range of adherence; min, max	Missing	*p*-value
Education level				1	0.040
low (secondary school or less)	30	18	0, 40		
medium (high school)	74	28.5	0, 55		
high (university)	5	16	0, 29		
Work type					0.001
regular daytime work	30	31.5	5, 55		
shift work	72	21.4	0, 44		
other working time	8	33	2, 43		
Occupation					0.003
assistant nurse	43	16	0, 41		
nurse	56	28	0, 55		
other (radiographer, PT, midwife)	11	35	4, 50		
Sub-study					0.012
Nurse I	27	12	0, 39		
Nurse II	41	24	0, 50		
Nurse III	42	29	0, 55		
Perceived health in comparison to others of the same age and gender					0.037
moderate	45	22	0, 55		
good or very good	65	28	0, 50		
Perceived fitness in comparison to others of the same age and gender					0.06
worse	32	23	0, 55		
equal	52	22	0, 44		
better	26	31	0, 55		
Frequency of LBP				8	0.051
on some days of the week	46	22	0, 43		
on most days	38	29	0, 55		
daily	18	40	0, 42		

*LBP* low back pain, *PT* physiotherapist

**Table 3:** Baseline variables of the participants (randomised to the exercise group) by exercise adherence status

	Compliers (≥24 exercise sessions), *n* = 58			Non-compliers (0–23 exercise sessions), n = 52			Miss-ing	*p*-value
	Mean (SD)	*n*	%	Mean (SD)	n	%		
Running figure-of-eight; seconds	7.7 (1.0)			8.0 (1.2)			9	0.20
One-legged squat; (0–12 reps)	9.9 (2.3)			8.9 (2.9)			2	0.043
6MWT; metres	623.0 (43.8)			603.4 (56.2)			–	0.048
Quality of life								
Physical functioning (0–100)	87.3(11.1)			83.4 (13.4)			4	0.17
General health (0–100) *	70.2(16.4)			64.5 (17.5)			4	0.08
Workability index (3–27)	22.2 (2.6)			21.9 (2.9)				0.20
PHQ-9 (0–27)	7.4 (4.5)			8.5 (5.3)			1	0.29
Musculoskeletal exertion (7–35) *	12.2 (3.8)			13.5(4.0)			2	0.10
FABs total (0–78)	23.2 (12.9)			27.3(14.5)			7	0.07
FAB-PA; (0–30)	12.6(6.9)			15.4 (6.4)			1	0.019
LBP intensity; (VAS 0–100)	36.9 (19.9)			35.9 (19.9)			1	0.79
Education level							–	0.026
low (secondary school or less)		14	24.1		23	44.2		
medium or high		44	75.9		29	55.8		
Work type							–	0.023
regular work		24	41.4		11	21.2		
shift work		34	58.6		41	78.8		
Profession							–	0.052
assistant nurse		18	31.0		25	48.1		
nurse		31	53.4		25	48.1		
other (radiographer, PT, midwife)		9	15.5		2	3.8		
Sub-study							–	0.042
Sub-study I		9	15.5		18	34.6		
Sub-study II		22	37.9		19	36.5		
Sub-study III		27	46.6		15	28.8		
Perceived health in comparison to others of the same age and sex; °							–	0.14
moderate		20	34.5		25	48.1		
good or very good		38	65.5		27	51.8		
Frequency of LBP; °							8	0.12
on some days of the week		21	38		25	54		
on most days		26	46		12	26		
daily		9	16		9	20		

*normal distribution, independent samples *t*-test, Mann–Whitney *U* test, ° χ^2^ test. *FAB-PA* physical activity-related fear-avoidance beliefs, *LBP* low back pain, *PHQ-9* Nine-item Patient Health Questionnaire measuring depression, *PT* physical therapist, *VAS* visual analogue scale of 0–100 during the past 4 weeks

Exercise non-compliers more often had a lower education level (*p* = 0.03) and did shift work (*p* = 0.02) compared to the exercise compliers (Table 3). From the baseline variables, higher fitness results in the one-legged squat (*p* = 0.043) and 6-min walk test (*p* = 0.048) were detected for exercise compliers (Table 3).

### Fear-avoidance beliefs

At the baseline, there was a difference in the levels of FABs between occupational groups in the whole study sample (*n* = 219). Nursing assistants had more FABs related to physical activity (FAB-PA, mean 15.5, SD 6.0, *n* = 89) than nurses (12.0, SD 5.9, *n* = 102) and other professionals (11.6, SD 6.8, *n* = 28) (F = 9.5, *p* < 0.001).

**Fig. 2:**
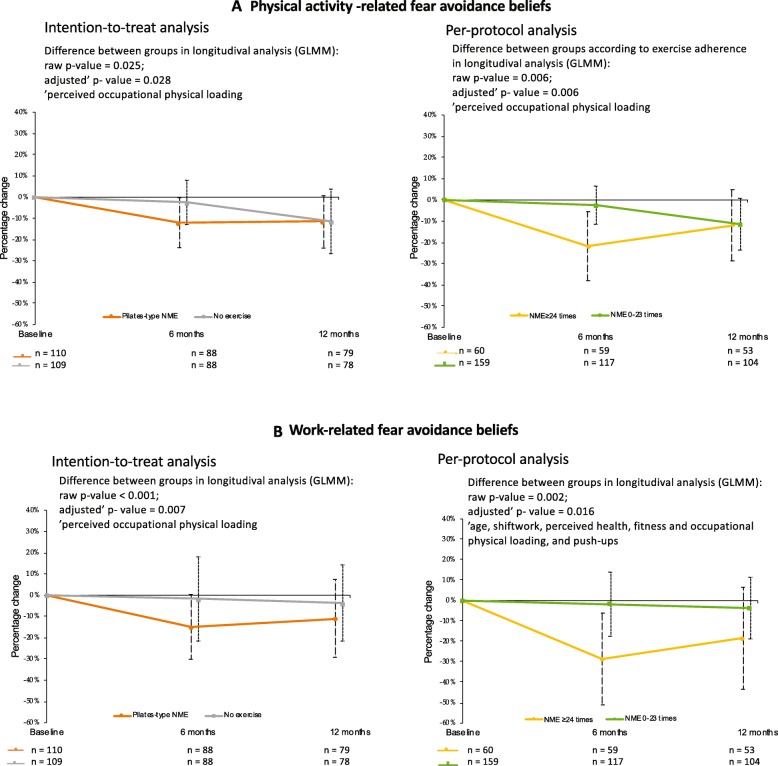
Effects of the exercise program on fear avoidance beliefs. Footnote to Fig. 2: Effects and effectiveness of the modified Pilates-type neuromuscular exercise (NME) with a focus on controlling the neutral spine on **a** physical activity-related fear-avoidance beliefs, and **b** work-related fear-avoidance beliefs (the mean difference in percentage with 95% confidence intervals analysed by generalised linear mixed models)

Exercise compliers showed lower values for FAB-PA at the baseline compared to non-compliers (*p* = 0.02, Table 3). During the exercise intervention, both FAB-PA (*p* = 0.028, adjusted for perceived occupational physical loading) and also FAB-W (*p* = 0.007, adjusted for age, shift work, perceived health, fitness and occupational physical loading, and push-ups) decreased in the exercise group compared to the non-exercisers (Fig. 2; ITT analysis). There was a dose-response; both FAB-PA (*p* = 0.006) and FAB-W (*p* = 0.016) decreased more in the high exercise adherence group compared to the less exercised and non-exercisers (Fig. 2; PP analysis). At 12 months follow-up, there were no more group differences in FAB-PA. A reduction in FAB-PA (from the baseline to 6 months) did not correlate with a reduction in LBP intensity (r_p_ = 0.03, *p* = 0.54), but there was a correlation between a reduction in FAB-W and a reduction in LBP intensity (r_s_ = 0.16, *p* = 0.05).

## Discussion

In this 6-month modified, Pilates-type exercise study for female healthcare personnel with sub-acute or recurrent, non-specific LBP, those possessing a lower basic education level, working shifts, and having lower levels of fitness and higher levels of physical activity-related FABs at the baseline had a lower exercise adherence. Exercising during the intervention reduced levels of FAB-PA and FAB-W, and there was a dose-response: the levels of FAB-PA and FAB-W decreased more in more exercised persons.

In exercise interventions, levels of exercise adherence usually drop over time; approximately 50% reduction in 12 months has been presented [26, 53]. In the present intervention, participation in the supervised groups decreased across time, but the amount of home-based exercise increased commensurately. The exercise videos, booklets, supportive e-mails and letters probably helped in maintaining the same exercise adherence level throughout the 6-month intervention. While we knew that exercise adherence is usually only modest at best among people with musculoskeletal pain [20], and that shift work makes attending regular group-based exercise demanding [54], the exercise adherence rate in the present study was lower than we expected. In 2012–2014, when the study interventions were conducted, Pilates was quite a popular exercise type in Finland, and for that reason it was expected to be more attractive than a typically conventional/traditional neuromuscular exercise form.

It has been suggested that factors associated with exercise adherence among LBP patients can be divided into three categories [55]: 1) physical factors like pain [55] and perceived health status [56]; 2) psychological factors such as the fear of pain [23], diagnostic uncertainty [22], low self-efficacy [21], and depression and anxiety [21]; and 3) environmental factors, such as difficulty in integrating exercise into daily life [22, 55], lack of time [22, 23, 55], and intervention-related variables [33]. This classification is partly insufficient: it is difficult to place education level, which is a socio-demographic background factor, into any of those categories. Socio-economic status is associated with back-related beliefs: those with high socio-economic status are more prone to believe that one should stay active regardless LBP [57]. Education level has been shown to affect adherence to exercise progression among people with chronic LBP [58], strength training [56], and leisure-time physical activity [59]. In the present study, lower basic education level was also associated with lower exercise adherence, even though healthcare workers are a fairly homogenous group, and socio-demographic differences are generally small in Finland [60].

In previous studies, LBP intensity [55] and older age [58] compromised adherence to exercise or exercise progression. Contradictory to earlier studies, they had no effect in the present study, perhaps because in most participants the intensity of LBP was mild to moderate, they were still working, and the age range was set to 30–55 years. Higher physical fitness level at the baseline contributed to better exercise adherence. To our knowledge, association between baseline physical fitness and exercise adherence in later intervention has not been reported previously.

FABs-PA results at baseline in the current study were comparable to those detected among French hospital workers with recurrent LBP [61], but lower than among LBP patients seeking medical care for their pain [62]. FABs related to physical activity are known to affect exercise adherence [22]; activities or exercises are avoided for fear of increasing pain [23]. Cognitive and psychological interventions [27] and graded activity [63] are usually considered helpful in the management of fear avoidance-related pain. An intervention including education in addition to exercise was slightly more effective in reducing FABs-PA among French hospital workers with LBP than the present study [61]. Interventions to reduce FABs very seldom include exercise only, but a Pilates-type exercise has shown to reduce FABs more than stationary cycling in short term among chronic LBP patients [64]. We hypothesise that this modified, slowly progressing Pilates-type exercise programme, which was conducted at the pace of the participant’s calm breathing tempo, might have given the participants positive experiences of movement. They could move in a way that they could control; the movements were not harmful or dangerous and could even release pain. This might explain the reduction of FAB levels during the exercise intervention.

*Moderators* (or treatment effect modifiers) are baseline characteristics that influence the outcome of treatment [65]. *Mediators* are factors that change during or as a consequence of an intervention and thereby influence outcome [65]. Thus, it might be hypothesised that the earlier reported reduction of LBP intensity among the present study sample after exercise intervention [34] might have been mediated by a reduction in the fear of movement. Nevertheless, there was no correlation between the reduction of LBP intensity and the reduction of FAB-PA, but exercise adherence was the key factor. Those with a lower FAB-PA at the baseline exercised more, and those who exercised more gained more positive results in pain reduction [34]. While we measured pain intensity only at the baseline and after the 6-month exercise intervention – i.e. not during the intervention period – we cannot say anything about causality. Exercising more might have reduced the pain levels due to exercise-induced hypoalgesia [66–68], or a potential rapid reduction of pain intensity at the beginning of the exercise intervention might have decreased the levels of FAB-PA, and thus increased the motivation to exercise. In exercise intervention, both FABs [69] and exercise adherence [26] can mediate the outcome, i.e. LBP intensity. Identification of the mechanisms through which different treatments affect outcomes is complicated, and there is a clear need for further research that investigates plausible mediators [69].

The reduction of FABs during the intervention was statistically significant, but we do not know its clinical relevance (three questions related to returning to work were removed from the original FAB Questionnaire). Among hospital workers with recurrent LBP, an intervention combining education and exercise reduced FABs-PA, but did not reduce LBP recurrence episodes in two-year follow up [61]. For those healthcare workers with previous LBP, both the physical workload and FABs are important in the development of new episodes of LBP [70].

A lack of time is the most frequently reported barrier to leisure-time physical activity or exercise, both among the general population [31, 71] and among people with LBP [23]. Those working shifts had a lower exercise adherence than those working at regular times. Among nursing personnel, shift work is also associated with sleeping problems, fatigue, and lack of energy [72, 73], which might compromise exercise adherence.

Increasing adherence to exercise is an important factor for the longer-term effectiveness of an intervention. Integrating educational components to exercise sessions, like strategic planning [23], self-monitoring [56], goal-setting [25, 74], supplementary printed material, motivation strategies and positive re-enforcement [74], encouragement and action planning to overcome barriers to exercise [23] have been suggested to increase exercise adherence. Also leadership and organisation skills [21], favourable environment and pleasure associated with exercise [23], and appropriate intensity of the training content [75] might help in reducing fear of pain and pain itself [23] and thus increase exercise adherence. Identification of especially those who have a low education level, and targeting motivating efforts at them [56] might be effective.

Understanding the causality and reasons for exercise adherence is complicated, multidimensional, and difficult to study. People do not always behave in the way they intend to behave. Motivation alone is not sufficient to trigger an action, and one is often confronted with obstacles [23]. In the present study, exercise adherence was lower among those with a lower level of basic education. The levels of baseline FABs were also higher among assistant nurses. In clinical practice, motivational strategies with a focus on decreasing FABs especially among people with a low education level could be beneficial. Unfortunately, there is a lack of measurement methods to identify those who would benefit most from motivational actions.

### Limitations

This study was a secondary analysis of the NURSE-RCT. Investigating associations between individual factors at the baseline and exercise adherence was not planned simultaneously with planning the RCT, and it was not written into the study protocol [35]. We arrived at the idea for the study after we detected the dose response of exercising on LBP intensity and movement control impairments [34]. Due to the four-arm setting of the original NURSE-RCT (combined exercise + counselling, exercise only, counselling only, controls), targeting motivational strategies at exercisers (exercise only and the combined group) would have been difficult without contaminating the back care counselling intervention.

Several additional measurements might have been beneficial: the immediate effects of the exercise sessions (to pain or other bodily sensations), home environment, previous physical activity (in earlier years), and the number and ages of the participants’ children were not ascertained in the study. This might have broadened our understanding of the factors affecting exercise adherence. We measured only the number of exercise sessions, which were either supervisor-documented (for group sessions) or self-reported (for home practice). The research calls for standard validated measures of exercise adherence [20].

## Conclusion

Participants with lower education and fitness levels who worked shifts and had high physical activity-related fear-avoidance beliefs at the baseline had a lower adherence to the 6-month neuromuscular exercise programme. Exercising with good adherence reduced levels of FABs, which are known to be linked with prolonged LBP. In exercise interventions, motivational strategies should be targeted at those with low education and fitness levels and high fear-avoidance beliefs to achieve better exercise adherence. In exercise intervention studies, strategies to enhance and/or maintain exercise adherence need to be taken more seriously, because adherence is a key link between intervention and outcomes.

## Data Availability

The datasets used and analysed during the current study are available from the principal researcher (JS; jaana.suni@ukkinstituutti.fi) of the NURSE RCT and from tommi.vasankari@ukkinstituutti.fi upon reasonable request.

## Abbreviations

ANOVA: Analysis of variance
BMI: Body mass index
DVD: Digital video disc
FABs: Fear-avoidance beliefs
FAB-PA: Fear-avoidance beliefs related to physical activity
FAB-W: Work-related fear-avoidance beliefs
GLMM: Generalised linear mixed model
ITT: Intention to treat
LBP: Low back pain
NME: Neuromuscular exercise
NRS: Numeric rating scale; 0–10
PHQ-9: Patient health questionnaire, 9 items
PP: Per protocol
RCT: Randomised controlled trial
SD: Standard deviation
VAS: Visual analogue scale; 0–100
